# Functional shifts in bird communities from semi-natural oak forests to conifer plantations are not consistent across Europe

**DOI:** 10.1371/journal.pone.0220155

**Published:** 2019-07-22

**Authors:** Scott M. Pedley, Luc Barbaro, João L. Guilherme, Sandra Irwin, John O’Halloran, Vânia Proença, Martin J. P. Sullivan

**Affiliations:** 1 School of Science and the Environment, Manchester Metropolitan University, Manchester, United Kingdom; 2 Dynafor, INPT, EI Purpan, INRA, Univ. Toulouse, Auzeville, France; 3 Centre d'Ecologie et des Sciences de la Conservation, Muséum national d’Histoire naturelle, CNRS, Sorbonne Université, Paris, France; 4 German Centre for Integrative Biodiversity Research (iDiv) Halle-Jena-Leipzig, Leipzig, Germany; 5 Institute of Biology, Martin Luther University Halle-Wittenberg, Halle (Saale), Germany; 6 School of Biological, Earth and Environmental Sciences, University College Cork, Cork, Ireland; 7 MARETEC, Marine, Environment and Technology Centre, Instituto Superior Técnico, Universidade de Lisboa, Lisboa, Portugal; 8 School of Geography, University of Leeds, Leeds, United Kingdom; Michigan State University, UNITED STATES

## Abstract

While the area of plantation forest increased globally between 2010 and 2015, more than twice the area of natural forests was lost over the same period (6.5 million ha natural forest lost per year versus 3.2 million ha plantation gained per year). Consequently, there is an increasing need to understand how plantation land use affects biodiversity. The relative conservation value of plantation forests is context dependent, being influenced by previous land use, management regimes and landscape composition. What is less well understood, and of importance to conservation management, is the consistency of diversity patterns across regions, and the degree to which useful generalisations can be provided within and among bioregions. Here, we analyse forest birds in Ireland, France and Portugal, representing distinct regions across the Atlantic biogeographic area of Europe. We compared taxonomic, functional and phylogenetic diversity of bird communities among conifer plantations and semi-natural oak forests, and assessed correlations between species traits and forest type across these regions. Although bird composition (assessed with NMDS ordination) differed consistently between plantation and oak forests across all three regions, species richness and Shannon diversity did not show a consistent pattern. In Ireland and France, metrics of taxonomic diversity (richness and Shannon diversity), functional diversity, functional dispersion and phylogenetic diversity were greater in oak forests than plantations. However, in Portugal taxonomic and phylogenetic diversity did not differ significantly between forest types, while functional diversity and dispersion were statistically significantly greater in plantations. No single bird trait-forest type association correlated in a consistent direction across the three study regions. Trait associations for the French bird communities appeared intermediate between those in Ireland and Portugal, and when trait correlations were significant in both Ireland and Portugal, the direction of the correlation was always opposite. The variation in response of bird communities to conifer plantations indicates that care is needed when generalising patterns of community diversity and assembly mechanisms across regions.

## Introduction

Globally, the area of natural and semi-natural forests (the latter being forests with predominantly natural characteristics but some human influence, such as historic management), decreased by 6.5 million hectares per year between 2010 and 2015 [1]. In contrast, the area of planted forest increased by 3.2 million hectares per year over the same period [1]. Understanding the degree to which plantation forests can sustain native forest-dependent species is thus critical to inform conservation management [2–6].

Plantation forests differ from semi-natural forests in several ways, leading to important differences in diversity and community composition across a variety of taxonomic groups and within many biogeographical regions [7–10]. In general, plantation forests are grown for wood extraction, typically resulting in large monoculture forests of uniform age and size-classes, and composed of fast-growing tree species placed evenly at high densities. Furthermore, plantation forests are typically subject to short to mid-rotation cycles involving the clearing of entire stands at coarse scales. These features contribute to generally lower vertical structure, from simpler canopies to reduced ground flora and understory [11–13]. Nevertheless, these differences may be contextual and related to previous land use, plantation management and land use in adjacent plots, and may vary across scales [3, 14].

Forest bird communities may therefore be particularly affected by the transition of semi-natural forests to plantation forests. The marked differences between these forest types strongly affect the availability of key resources [12, 15, 16], and reduce important behaviours like foraging success, nesting ability and predator avoidance [17, 18], resulting in depauperate communities with significantly less species [13, 14, 19]. For example, Sweeney et al. [13] showed that non-native Sitka spruce (*Picea sitchensis*) plantations in Ireland had significantly fewer bird species and dissimilar community composition than native semi-natural forests. Beyond the negative effect on species richness, both bird phylogenetic and functional trait diversity can be modified by the replacement of native semi-natural forests by fast-growing plantation forests, which may affect the provision of both regulating and cultural (e.g. recreational birdwatching) ecosystem services by native bird communities [19, 20].

Relating responses of individual species to their functional and life-history traits (e.g. [21, 22]) permits a deeper understanding of the mechanisms causing community level responses to environmental disturbances [23], such as a shift to plantation forests, while providing insights into the mechanisms influencing species-specific responses [24]. However, there is little understanding of how predictable these differences might be across biogeographical regions, and attempts to assess if findings from one region can be generalized to other regions are still rare. One exception is an investigation into responses of farmland passerines to agricultural intensification [25], which found that models constructed in one region had lower predictive power when applied to other regions. If trait-driven species responses to forest management are consistent across regions, potential trait redundancies (where traits are shared by multiple species in a community) or complementarities (where species in a community have unique traits), likely to impact the provisioning of ecosystem services by forest birds, could be established [26, 27]. Conversely, if regional variation in species trait-forest type relationships are widespread, then inferences on bird community responses will not be predictable across regions. Nevertheless, variation may reveal regional-specific effects that modulate such species and community responses, thus enhancing our understanding to inform regional forest management.

To assess if bird community-forest type relationships are consistent between regions, we compare bird communities in semi-natural oak (*Quercus* spp) forests and conifer plantations, and use this spatial variation to evaluate differential bird responses to these two types of forest. To assess whether these responses are consistent among regions, we compiled data from three regions (Ireland, France and Portugal) representing distinct Atlantic biogeographic areas of Europe. Specifically, we examined (a) if bird community composition and diversity (taxonomical, functional and phylogenetic) in conifer plantations differed from semi-natural forests in each study region, (b) if these responses were consistent across study regions, and (c) whether consistent trait-environment relationships determined which species responded positively or negatively to plantation forests.

## Materials and methods

### Study regions and data harmonization

We recorded forest bird communities from three study regions located in Ireland, France and Portugal, within the Atlantic biogeographical region of Western Europe (see S1 Table for regional environmental characteristics). Data for each study region were originally collected as part of independent studies [13, 28, 29], hence, for consistency among datasets we restricted the original datasets to only two forest types, extracting the data from closed-canopy monoculture stands composed of even-aged native and non-native conifer species (hereafter conifer plantations), and from mature native semi-natural deciduous forests dominated by oak species (hereafter semi-natural oak forests). Semi-natural oak forests constitute the climax vegetation of the three study regions and are currently under reduced or no management. Although these semi-natural oak forests have been influenced by differing disturbances (e.g. fire, firewood collection) in the past, current structural and microclimatic conditions provide useful comparative systems. The selection of these two forest types resulted in ten conifer patches and seven semi-natural oak forest patches in Ireland, 64 conifer patches and 40 semi-natural oak forest patches in France, and nine conifer patches and nine semi-natural oak forest patches in Portugal. In Ireland, the forests studied were located across the south of the island, where conifer plantations consisted of non-native Sitka spruce with rotation cycles of 30–50 years. Sitka spruce is the dominant species in commercial forests in Ireland, accounting for 60% of the plantation estate. Semi-natural oak forests were dominated by Pedunculate oak (*Quercus robur*) and Sessile oak (*Q*. *petraea*), but also comprised Downy birch (*Betula pubescens*) and Holly (*Ilex aquifolium*). In France, the study area was located in the southwest region of Aquitaine, the largest area of pine plantations in the country. Landscapes are dominated by mosaic plantations of even-aged stands of native Maritime pine (*Pinus pinaster*) with rotation cycles of 40–50 years; these plantations are interspersed with open habitats and fragments of semi-natural oak forest dominated by Pedunculate oak, Pyrenean oak (*Q*. *pyrenaica*) and Silver birch (*B*. *pedula*). In Portugal, the study sites were located in the Alto Minho region, in the northwest of the country, in a mosaic landscape of forest, agriculture and scrubland. Surveyed semi-natural oak forests were dominated by Pedunculate oak and Pyrenean oak, but also included Iberian Downy birch (*B*. *celtiberica*). Conifer plantations were monocultures of the native Maritime pine with rotation cycles of 20–45 years; this species is one of the most dominant commercial species in Portuguese planted forests, especially in northern regions. Although Maritime pine is a native species to Portugal and France, its natural distribution is unclear as a result of extensive commercial exploitation, which has significantly increased its distribution into areas where it may have not occurred naturally [30]. The area of forest patches was variable within each study region, but did not differ systematically between oak and conifer forest; in Ireland, all forest patches were > 6 ha, in France, both oak and conifer patches varied from 1 ha to 25 ha, and in Portugal oak and conifer patches varied from < 1ha to 31 ha.

In all three studies, we used point counts to record bird communities [13, 28, 29], but due to the different survey designs and survey efforts employed in the original studies, it was not possible to pool the three data sets. Instead, we ran separate analyses for each region to examine whether bird communities differed between forest types, and then compared qualitatively the consistency of these differences among regions. Species for which the point count survey protocol was not appropriate to assess abundance, such as nocturnal species where daytime observations would be highly influenced by chance, were excluded before analysis (see S1 File for details on point count sampling designs and excluded species).

### Analysis

All analyses were carried out using R [31]. To visualize the sampled community composition in semi-natural forests and conifer plantations, we used non-Metric Multidimensional Scaling (NMDS), performed on a matrix of Bray-Curtis dissimilarities of abundance data (square root transformed and Wisconsin double standardization) using the ‘vegan’ package [32]. Differences in community composition between forest types were tested using the ‘mvabund’ package [33], which allows hypothesis testing by multivariate implementation of Poisson generalized linear models; we tested for significant differences in assemblage composition of conifer plantations versus semi-natural oak forests using likelihood-ratio-tests.

### Taxonomic and functional diversity

Functional traits for the 59 bird species recorded in the dataset (S2 Table) were used to calculate functional diversity metrics (Rao’s quadratic entropy, which is a modification of Simpson’s diversity index to incorporate functional differences between species, and functional dispersion, which represents the functional dissimilarity among species in a community) across the three study regions, using the ‘FD’ package [34]. We selected species traits on the basis of their expected role in shaping species responses to different environments [35] (i.e. semi-natural forest and conifer plantation). Specifically, we selected response-mediated traits regarding (1) body mass, (2) resource use and acquisition (diet type, main foraging substrate and bill length), (3) habitat specialization (habitat affinity and nesting location), (4) reproductive effort (clutch size), (5) life span, (6) migratory status, and (7) home range-size during the breeding season. All trait information was available at the individual species level from published literature [36, 37]. Traits were organized into binary dummy variables for testing directly the trait response to environmental condition (i.e. forest type); see S2 Table for a full list of the trait sub-categories, descriptions and data sources. In addition, we also calculated two taxonomic metrics used commonly in ecological studies: species richness that measures only species incident, and Shannon diversity index that also incorporates species abundance.

### Phylogenetic diversity

We obtained subsets of a global phylogeny [38], and subset to only include species recorded in our dataset. We downloaded 1000 phylogenies from the posterior distribution of the version of this phylogeny constrained to the Ericson All Species backbone, available from www.birdtree.org. We calculated phylogenetic diversity as the sum of branch lengths of the subset of the phylogeny containing all species in a community using the PD function in the ‘picante’ package [39]. We calculated phylogenetic dispersion (*D*) using the phylo.d function in the ‘caper’ package [40]; *D* assesses phylogenetic signal in binary traits (such as community membership) against null models of Brownian motion trait evolution and phylogenetic randomness, and is scaled to be zero under Brownian motion trait evolution and one under phylogenetic randomness [41]. Only species recorded in each study region were used in the source pool phylogeny for calculating *D*. We calculated phylogenetic diversity and dispersion for each forest patch and phylogeny, before taking the mean value across phylogenies for each study region.

### Diversity analysis

All taxonomic, functional and phylogenetic metrics (see above) were calculated at the forest-patch level, before taking the mean value across each forest type and across each study region. As the majority of metrics calculated had non-normal distributions, we used non-parametric means tests (Mann-Whitney U) to identify differences between semi-natural oak forests and conifer plantations.

### Linking bird traits to forest types

Trait-specific responses to conifer plantations were analysed by means of fourth-corner analysis, using the ‘ade4’ package [42, 43]. Fourth-corner analysis directly tests the link between all combinations of species traits and environmental attributes (i.e. forest type: semi-natural oak forests or conifer plantations). The procedure uses three data-tables where matrix ‘R’ (environmental attributes versus site) is indirectly related to matrix ‘Q’ (trait versus species), via a third matrix ‘L’ (species abundance versus site). Using a generalized statistic S_RLQ_, the fourth-corner procedure can analyse quantitative variables, qualitative variables or a mixture of both. We used binary coding of the environment matrix (forest type) against a quantitative and binary trait matrix resulting in the generalized statistic being equal to the Pearson correlation coefficient r. Binary coding also allowed for directional correlations (positive or negative) between species traits and forest types. We applied permutation Model 1 (with 9999 permutations) to test the null hypothesis that R is not linked to Q, when examining links between the fixed table of species traits and the fixed table of site attributes, mediated by the observed abundance data of matrix L [42]. We opted for Model 1 because it randomises presence-absence of individual species relative to site characteristics (permuting within each column of matrix L), without re-sampling the species-trait relationship (matrix Q) or the environment-site relationship (matrix R). This was the appropriate approach for our dataset as traits were determined from the literature and not by empirical sampling, and the environment attributes (forest type) were determined *a priori* [42]. We used false discovery rate correction procedures for multiple testing which, although potentially increases the number of type I errors, is a much more powerful approach to avoid misclassifying hypothesis that are statistically significant than traditional Bonferroni corrections [44].

## Results

### Taxonomic, functional and phylogenetic diversity

**Fig 1:**
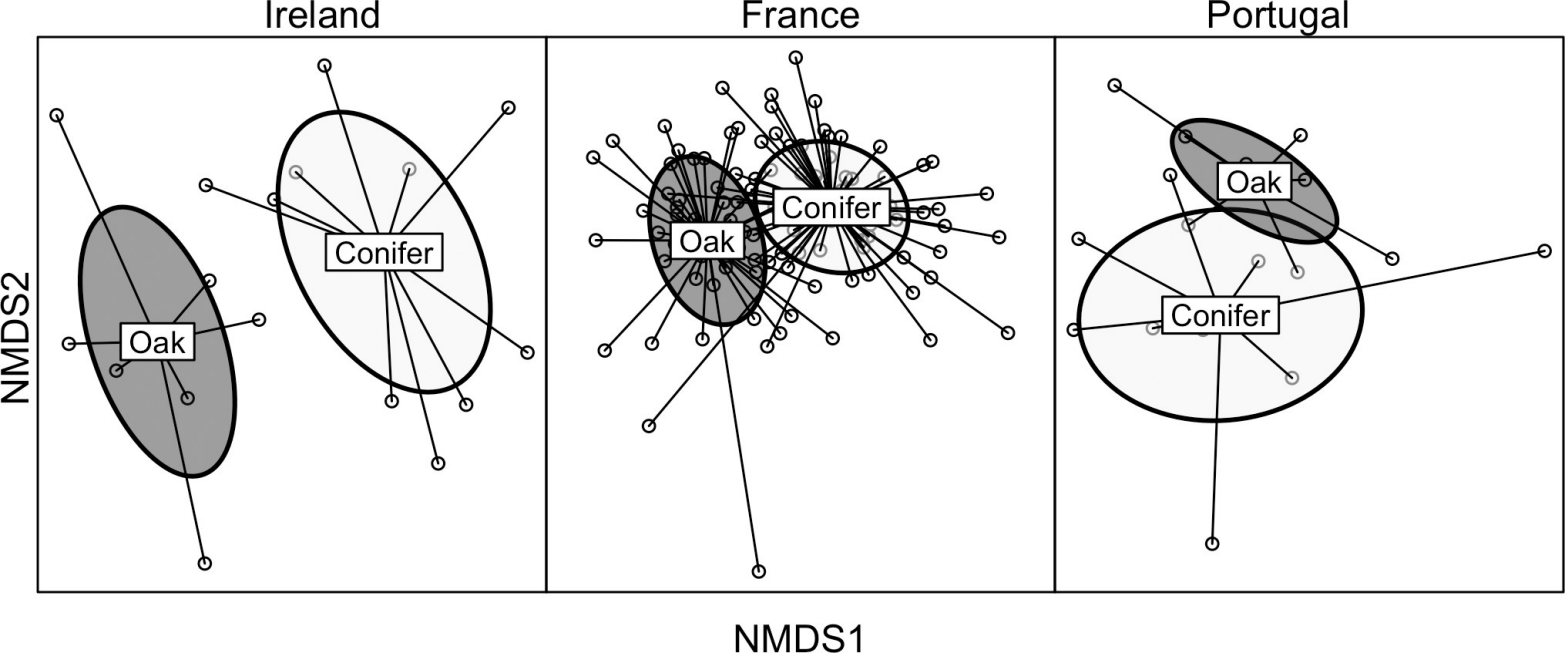
Non-Metric Multidimensional Scaling (NMDS) ordination comparing bird assemblage composition among semi-natural oak forests and conifer plantations in Ireland, France and Portugal (stress scores: Ireland = 0.197, France = 0.222, Portugal = 0.161). Points are forest patches with lines connecting to forest-type centroids. Ellipses represent the standard deviation of forest-type centroids.

Significant differences in bird community composition were found between conifer plantations and semi-natural oak forests in all regions sampled (Ireland: deviance = 207.6, P = 0.002; France: deviance = 465.1, P = 0.001; Portugal: deviance = 59.87, P = 0.015). Community composition in Irish assemblages showed greater separation between forest types, while the overlap in forest type ellipses was more obvious in the Portuguese assemblages, and intermediate in France (Fig 1).

**Fig 2:**
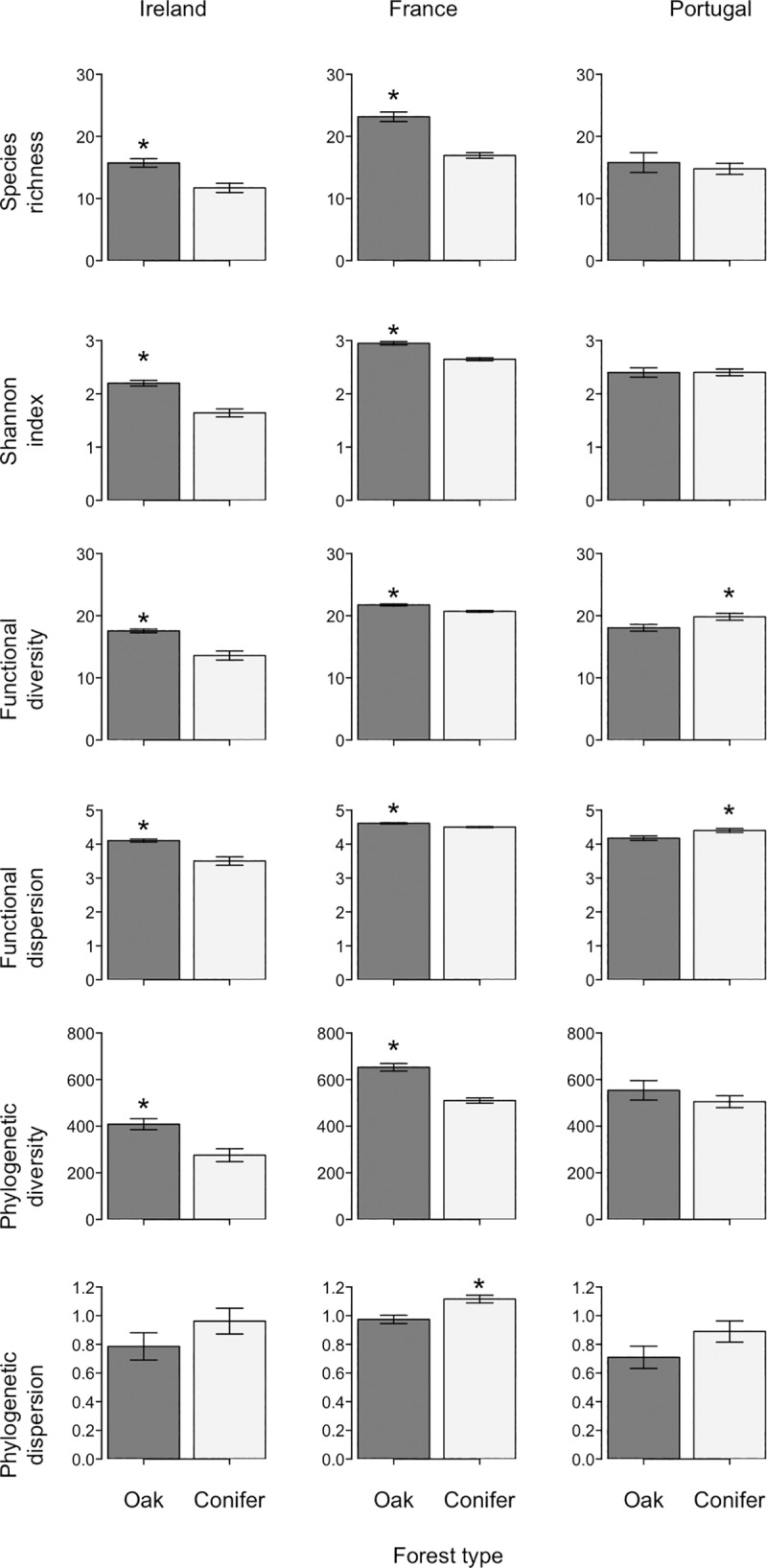
Responses of bird assemblages to semi-natural oak forests and conifer plantations in Ireland, France and Portugal. For each richness, diversity and dispersion metric the mean value and the standard error are shown; asterisk denotes significant differences (Mann-Whitney U test, P<0.01) between the two forest types (see S4 Table for test statistics).

Responses of bird communities to conifer plantations were inconsistent between Ireland and Portugal. In Ireland, species richness and Shannon diversity were significantly lower in conifer plantations than in semi-natural oak forests (richness: W = 65, P = 0.004; Shannon: W = 70, P<0.001), while bird communities were also less functionally diverse and clustered in functional space (Functional diversity: W = 70, P<0.001; Functional dispersion: W = 70, P<0.001; Fig 2). These differences were also evident in France (richness: W = 2210, P<0.001; Shannon W = 2169, P<0.001), however, differences in mean functional diversity and dispersion were much smaller than in Ireland (Functional diversity: W = 1931, P<0.001; Functional dispersion: W = 1900, P<0.001; Fig 2). In Portugal, species richness and Shannon diversity were similar between conifer plantations and semi-natural oak forest, and in contrast to the other two regions, conifer plantations had greater functional diversity and dispersion than semi-natural oak forests (Functional diversity: W = 16, P = 0.031; Functional dispersion: W = 15, P = 0.024, Fig 2).

Phylogenetic diversity was lower in conifer plantations than in semi-natural oak forests in both Ireland (W = 64, P = 0.003) and in France (W = 2176, P<0.001), but did not differ significantly between these forest types in Portugal (Fig 2). Phylogenetic dispersion was higher in conifer plantations than semi-natural oak forests in all regions but only significantly so in France (W = 763, P = 0.001).

### Trait-environment associations

No single trait-forest type association correlated in a consistent direction across all three study regions (Table 1 and S3 Table). Again, results from France were intermediate between those in Ireland and Portugal. That is, response patterns were either shared with Ireland or with Portugal but never with both regions simultaneously. Where trait correlations were significant in both Ireland and Portugal, the sign (direction) of the correlation was always opposite (Table 1). The only consistent directional correlation for France and Portugal was for small/medium clutch sizes. There were a greater number of significant trait correlations in France than in Ireland or Portugal, although sample size and hence statistical power was greater for the French dataset.

**Table 1:** Species trait relationships within conifer plantation forests relative to semi-natural oak forests in Ireland, France and Portugal, as calculated with fourth-corner correlations between traits and environmental variables (see S3 Table for fourth corner test statistics).

		Conifer plantation forest		
Trait group	Traits	Ireland	France	Portugal
Body size	mass	**-**	**-**	**+**
Diet	herbivore		**-**	**+**
	insectivore		**+**	
	mixed		**-**	
Foraging	air	**-**	**-**	
	ground			
	lower vegetation		**+**	
	upper vegetation			**-**
	mixed	**-**		**+**
Bill length	average		**-**	**+**
Habitat	specialist		**+**	**-**
Nest location	cavity		**-**	
	ground		**+**	
	shrub			**+**
	tree			
	mixed	**-**	**-**	
Clutch size	small/medium	**-**	**+**	**+**
Life span	short	**+**	**+**	
Migration	migratory		**+**	
	resident	**+**		**-**
	mixed			**+**
Range	small	**+**		
	medium	**-**	**-**	
	large	** **	**+**	

In Ireland, small-bodied bird species with short life spans, large clutch sizes and small home ranges were associated with conifer plantations (Table 1). In France, species significantly associated with conifer plantations were insectivorous forest specialists with small body mass, low vegetation foraging strategy, ground located nests and relatively short life spans. In addition, migratory behaviour and large range sizes were also positively associated with conifer plantations. In contrast, traits associated with conifer plantations in Portugal were large herbivores with a mixed foraging strategy and preference for nesting in shrubs. Like the French bird assemblages, the ones found in Portuguese conifer plantations also had smaller clutch sizes; large bill sizes were also positively associated with conifer plantations in Portugal, as was a mixed migratory strategy.

## Discussion

### Regional congruence and divergence in bird community responses

In this study we examined whether taxonomic, functional and phylogenetic variation in bird assemblages between semi-natural forests and conifer plantations were congruent across a large latitudinal gradient in Western Europe. Although differences in bird communities between conifer plantations and native semi-natural oak forests were evident in all regions, responses to plantation forests were not consistent between Ireland, France and Portugal. In Ireland and France, taxonomic, functional and phylogenetic diversity were lower in conifer plantations than semi-natural oak forests, which is consistent with previous studies reporting significant decreases in species richness [45] and changes in functional composition [19] in plantation forests elsewhere in Europe. Interestingly, in Portugal diversity metrics showed a reverse pattern, with diversity either not statistically different between conifer plantations and semi-natural oak forest or greater in conifer plantations.

Differences in bird community responses to conifer plantations could result from broad-scale extrinsic factors such as climate, landscape and regional context [46] and bird assemblage source pool [47] of the study regions, or from factors intrinsic to the conifer plantations themselves (e.g. degree of forest management). One potentially important intrinsic factor is the different tree composition of the plantations in Ireland, which have monocultures of non-native Sitka spruce, and those in France and Portugal, which have monocultures of native Maritime Pine. Plantations composed of exotic species have been found to have lower bird species richness than those composed of native species [48], so the effect of plantations of bird communities might be expected to be greater in Ireland (Sitka spruce) than France and Portugal (Maritime Pine). Nevertheless, we argue that under active forestry management plantations of these two temperate conifer species are likely to display equivalent ecological conditions and comparable structure (i.e. even-aged monocultures with reduced understory) and resources for birds (e.g. nesting sites, foraging opportunities, and type of predators). Indeed, previous research has shown that in both Ireland and France, the age of the plantations, rather than tree species composition, is more likely to affect bird assemblages [49, 50]. Notably, the response of bird communities to conifer plantations between France and Portugal was as divergent as between those regions and Ireland, which suggests that tree composition may not be the key factor driving differences in bird responses to forest plantations.

Although it remains unclear which factors drive the differences observed between the three study regions, the lack of consistency in responses between regions indicates the importance of ecological contexts on bird community responses to conifer plantations. Therefore, we call for caution when attempting to generalize research findings from one location to another, or when scaling results up from regional to biogeographical scales.

### Consistency of trait effects among regions

Functional traits associated with bird species occurrence in conifer plantations differed among regions. For example, migratory bird species were positively associated with conifer plantations in France, while in Ireland it was resident species that were positively associated with conifer plantations. Again, it is not possible to definitively identify why trait associations varied from Ireland to Portugal, but we can propose some potential explanations for some traits. In Ireland, species with traits associated with fast life history strategies (i.e. small body size, short life-spans and large clutch sizes) and small home range size were positively associated with conifer plantations. Previous studies have demonstrated the importance of Sitka spruce plantations for bird species sharing this collection of traits (i.e. small body size, short life span), such as siskin (*Spinus spinus*) and coal tit (*Periparus ater*) [51]. Possibly, the generally low plant and invertebrate richness of conifer plantations in Ireland [9] affects bird communities through a lack of resources to support high abundances of large bird species. Associations with these traits were weaker in France, with only negative correlations with body mass and life-span remaining, while in Portugal large species with small clutches, represented by species such as Iberian green woodpecker (*Picus sharpei*) and golden oriole (*Oriolus oriolus*), were positively associated with conifer plantations. This pattern suggests that environmental filters acting in one region can be absent or reversed in another, such that the selection for small species in conifer plantations in Ireland was not observed in Portugal, where larger species were associated with conifer plantations.

The surrounding landscape context may influence the occurrence of birds in conifer plantations [52], so differences in the landscape context of conifer plantations amongst regions could lead to differences in the traits associated with conifer plantations. In the French study area, for example, cavity nesters typically associated with deciduous forests such as green woodpecker (*Picus viridis*), common redstart (*Phoenicurus phoenicurus*) and spotted flycatcher (*Muscicapa striata*), can nest in oak forest patches while foraging in nearby conifer stands. Conversely, conifer plantations in this region are often close or adjacent to open habitats (clearcuts, firebreaks, heathlands), which allows typical edge species such as tree pipit (*Anthus trivialis*), or species associated with young pine plantations such as grasshopper warbler (*Locustella naevia*) and Dartford warbler (*Sylvia undata*) to occur in older adjacent conifer plantations, especially along the forest edge [28, 49]. In Portugal, we found that species more associated with conifer plantations displayed functional attributes such as habitat generalism and mixed-foraging strategies. Conifer plantations in Portugal were within a diverse mosaic landscape with small urban areas, scrubland and patches of semi-natural forest interconnected by hedgerows bordering small-sized agricultural fields. These features allow birds to explore the wider landscape while moving among forest types, and possibly use resources available in different habitats [29, 53]. The role of surrounding landscape in influencing bird communities in conifer plantations is supported by previous work finding that the species richness of Maritime pine plantations in France and Portugal is higher when they have a greater edge extent and thus more opportunity for non-forest species to use forest habitat [29, 49].

## Conclusion

The responses of bird communities to conifer plantations, and the traits that determine which species were associated with plantations, varied among the three study regions. The inconsistent trait-environmental correlations among the three regions sampled suggest that functional generalizations across large geographical regions should be made cautiously. Although it was not possible to identify formally the contribution of intrinsic differences attributed to forest structure and management versus extrinsic differences attributed to landscape-scale factors, assemblage and climate; our work highlights that these processes can lead to context-dependent responses to conifer plantations. It is therefore important to capture these context-dependent processes if responses of birds to forestry are to be extrapolated confidently to new regions.

## Supporting information

S1 Table Environmental characteristics of the study regions. (DOCX)

S2 Table Bird species trait informaiton. (DOCX)

S3 Table Fourth-corner test statistics. (DOCX)

S4 Table Mann-Whitney U test statistics. (DOCX)

S1 File Point count sampling methodology. (DOCX)
