# Supplementary material for: Functional shifts in bird communities from semi-natural oak forests to conifer plantations are not consistent across Europe
Source: PLoS One. 2019 Jul 22;14(7):e0220155. doi: 10.1371/journal.pone.0220155 (PMC6645557; doi:10.1371/journal.pone.0220155)
Supplement: S1 Table — (DOCX) [file pone.0220155.s001.docx]

**S1 Table.** Environmental characteristics of the study regions. Climate variables were obtained for a centrally located grid-cell in each study area from the WorldClim database [1] (temperature and precipitation) or [2] for cumulative water deficit (CWD).

| Variable | Ireland | France | Portugal |
| --- | --- | --- | --- |
| Temperature* | 9.2 (4.9 – 14.1) | 12.8 (6.5 – 19) | 13.7 (7.9 – 19.7) |
| Precipitation** | 907 (178 – 269) | 1033 (195 – 338) | 1254 (89 – 497) |
| CWD | -40 | -182 | -339 |
| Semi-natural forest | *Quercus robur* and *Q. petraea* (n=7) | *Q. robur* and *Q. pyrenaica* (n=40) | *Q. robur* and *Q.* pyrenaica (n=9) |
| Plantation | Non-native *Picea sitchensis* (n=10) | Native *Pinus pinaster* (n=64) | Native P. pinaster (n=9) |

* Annual mean temperature values are presented, with values in the coldest and warmest quarter shown in parentheses.

** Total annual precipitation (with values for driest and wettest quarters in parentheses)

1. Hijmans RJ, Cameron SE, Parra JL, Jones PG, Jarvis A. Very high resolution interpolated climate surfaces for global land areas. Int J Climatol. 2005;25(15):1965-78.

2. Chave J, Rejou-Mechain M, Burquez A, Chidumayo E, Colgan MS, Delitti WB, et al. Improved allometric models to estimate the aboveground biomass of tropical trees. Global Change Biology. 2014;20(10):3177-90.
