# Supplementary material for: Functional shifts in bird communities from semi-natural oak forests to conifer plantations are not consistent across Europe
Source: PLoS One. 2019 Jul 22;14(7):e0220155. doi: 10.1371/journal.pone.0220155 (PMC6645557; doi:10.1371/journal.pone.0220155)
Supplement: S2 Table — (DOCX) [file pone.0220155.s002.docx]

**S2 Tables**: Bird species trait information

Table 1: Bird species recorded from each study region and associated traits organized into binary dummy variables. Also included is the proportion each species composed of the point count sample per region for each forest type.

|  |  | **Body size** | **Diet** | | | **Foraging** | | | | | **Bill length** | **Habitat** | **Nest location** | | | | | **Clutch size** | **Life span** | **Migration** | | | **Range** | | | **Proportion of sample** | |
| --- | --- | --- | --- | --- | --- | --- | --- | --- | --- | --- | --- | --- | --- | --- | --- | --- | --- | --- | --- | --- | --- | --- | --- | --- | --- | --- | --- |
| **Species** | **Region** | **mass** | **herbivore** | **insectivore** | **mixed** | **air** | **ground** | **low vegetation** | **upper vegetation** | **mixed** | **average** | **specialist** | **cavity** | **ground** | **shrub** | **tree** | **mixed** | **small/medium** | **short** | **migratory** | **resident** | **mixed** | **small** | **medium** | **large** | **Oak (%)** | **Conifer (%)** |
| *Aegithalos caudatus* | Ireland | 8.5 | 0 | 1 | 0 | 0 | 0 | 0 | 1 | 0 | 7.50 | 1 | 0 | 0 | 0 | 0 | 1 | 0 | 1 | 0 | 1 | 0 | 0 | 1 | 0 | 2.5 | 0.2 |
| *Spinus spinus* | Ireland | 13.8 | 1 | 0 | 0 | 0 | 0 | 0 | 0 | 1 | 12.95 | 0 | 0 | 0 | 0 | 1 | 0 | 1 | 1 | 0 | 0 | 1 | 0 | 0 | 1 | 0.2 | 0.1 |
| *Certhia familiaris* | Ireland | 8.905 | 0 | 1 | 0 | 0 | 0 | 0 | 1 | 0 | 16.85 | 1 | 1 | 0 | 0 | 0 | 0 | 1 | 1 | 0 | 1 | 0 | 0 | 0 | 1 | 2.8 | 0.3 |
| *Columba palumbus* | Ireland | 301 | 1 | 0 | 0 | 0 | 0 | 0 | 0 | 1 | 21.25 | 1 | 0 | 0 | 0 | 1 | 0 | 1 | 0 | 0 | 1 | 0 | 0 | 0 | 1 | 1.0 | 0.5 |
| *Corvus corone* | Ireland | 484 | 0 | 0 | 1 | 0 | 1 | 0 | 0 | 0 | 55.10 | 0 | 0 | 0 | 0 | 1 | 0 | 1 | 0 | 0 | 1 | 0 | 0 | 0 | 1 | 0.1 | 0.1 |
| *Corvus frugilegus* | Ireland | 462.75 | 0 | 0 | 1 | 0 | 1 | 0 | 0 | 0 | 59.65 | 0 | 0 | 0 | 0 | 1 | 0 | 1 | 0 | 0 | 1 | 0 | 0 | 0 | 1 | 0.0 | 0.0 |
| *Cyanistes caeruleus* | Ireland | 10.85 | 0 | 0 | 1 | 0 | 0 | 0 | 1 | 0 | 9.05 | 1 | 1 | 0 | 0 | 0 | 0 | 0 | 0 | 0 | 1 | 0 | 0 | 1 | 0 | 12.2 | 0.6 |
| *Erithacus rubecula* | Ireland | 18.25 | 0 | 0 | 1 | 0 | 1 | 0 | 0 | 0 | 14.30 | 0 | 0 | 1 | 0 | 0 | 0 | 1 | 1 | 0 | 1 | 0 | 1 | 0 | 0 | 19.9 | 15.3 |
| *Fringilla coelebs* | Ireland | 21.4 | 0 | 0 | 1 | 0 | 1 | 0 | 0 | 0 | 15.05 | 1 | 0 | 0 | 0 | 1 | 0 | 1 | 0 | 0 | 0 | 1 | 0 | 0 | 1 | 9.6 | 8.1 |
| *Garrulus glandarius* | Ireland | 161.7 | 0 | 0 | 1 | 0 | 0 | 0 | 0 | 1 | 33.20 | 1 | 0 | 0 | 0 | 1 | 0 | 1 | 0 | 0 | 1 | 0 | 0 | 0 | 1 | 0.8 | 0.5 |
| *Muscicapa striata* | Ireland | 14.7 | 0 | 1 | 0 | 1 | 0 | 0 | 0 | 0 | 15.45 | 0 | 0 | 0 | 0 | 0 | 1 | 1 | 1 | 1 | 0 | 0 | 1 | 0 | 0 | 0.2 | 0.0 |
| *Parus major* | Ireland | 17.75 | 0 | 0 | 1 | 0 | 0 | 0 | 1 | 0 | 12.55 | 1 | 1 | 0 | 0 | 0 | 0 | 0 | 0 | 0 | 1 | 0 | 0 | 1 | 0 | 1.5 | 0.3 |
| *Periparus ater* | Ireland | 9.7 | 0 | 0 | 1 | 0 | 0 | 0 | 1 | 0 | 11.15 | 1 | 1 | 0 | 0 | 0 | 0 | 0 | 1 | 0 | 1 | 0 | 1 | 0 | 0 | 13.5 | 25.6 |
| *Phylloscopus collybita* | Ireland | 7.7 | 0 | 1 | 0 | 0 | 0 | 0 | 1 | 0 | 11.10 | 1 | 0 | 1 | 0 | 0 | 0 | 1 | 1 | 1 | 0 | 0 | 0 | 0 | 1 | 1.2 | 0.6 |
| *Phylloscopus trochilus* | Ireland | 9.35 | 0 | 0 | 1 | 0 | 0 | 0 | 1 | 0 | 11.70 | 0 | 0 | 1 | 0 | 0 | 0 | 1 | 1 | 1 | 0 | 0 | 1 | 0 | 0 | 1.2 | 0.3 |
| *Pica pica* | Ireland | 213 | 0 | 0 | 1 | 0 | 1 | 0 | 0 | 0 | 40.30 | 0 | 0 | 0 | 0 | 1 | 0 | 1 | 0 | 0 | 1 | 0 | 0 | 0 | 1 | 0.1 | 0.1 |
| *Prunella modularis* | Ireland | 18.6 | 0 | 0 | 1 | 0 | 1 | 0 | 0 | 0 | 14.65 | 1 | 0 | 0 | 1 | 0 | 0 | 1 | 1 | 0 | 1 | 0 | 1 | 0 | 0 | 0.2 | 0.8 |
| *Pyrrhula pyrrhula* | Ireland | 21 | 1 | 0 | 0 | 0 | 0 | 0 | 1 | 0 | 15.35 | 1 | 0 | 0 | 0 | 1 | 0 | 1 | 1 | 0 | 1 | 0 | 0 | 1 | 0 | 0.3 | 0.0 |
| *Regulus regulus* | Ireland | 5.5 | 0 | 1 | 0 | 0 | 0 | 0 | 1 | 0 | 10.45 | 1 | 0 | 0 | 0 | 1 | 0 | 0 | 1 | 0 | 1 | 0 | 1 | 0 | 0 | 17.3 | 37.3 |
| *Sylvia atricapilla* | Ireland | 18.6 | 0 | 0 | 1 | 0 | 0 | 0 | 1 | 0 | 14.45 | 1 | 0 | 0 | 1 | 0 | 0 | 1 | 1 | 1 | 0 | 0 | 0 | 1 | 0 | 1.7 | 1.0 |
| *Sylvia borin* | Ireland | 19.05 | 0 | 0 | 1 | 0 | 0 | 1 | 0 | 0 | 14.50 | 1 | 0 | 0 | 1 | 0 | 0 | 1 | 1 | 1 | 0 | 0 | 1 | 0 | 0 | 0.0 | 0.0 |
| *Sylvia communis* | Ireland | 16.15 | 0 | 0 | 1 | 0 | 0 | 1 | 0 | 0 | 13.20 | 1 | 0 | 0 | 1 | 0 | 0 | 1 | 1 | 1 | 0 | 0 | 1 | 0 | 0 | 0.1 | 0.1 |
| *Troglodytes troglodytes* | Ireland | 8.9 | 0 | 1 | 0 | 0 | 0 | 1 | 0 | 0 | 13.65 | 0 | 0 | 1 | 0 | 0 | 0 | 1 | 1 | 0 | 1 | 0 | 1 | 0 | 0 | 6.4 | 5.6 |
| *Turdus merula* | Ireland | 95.85 | 0 | 0 | 1 | 0 | 0 | 0 | 0 | 1 | 17.20 | 0 | 0 | 0 | 1 | 0 | 0 | 1 | 0 | 0 | 0 | 1 | 0 | 1 | 0 | 5.4 | 1.8 |
| *Turdus philomelos* | Ireland | 70.5 | 0 | 0 | 1 | 0 | 1 | 0 | 0 | 0 | 14.25 | 0 | 0 | 0 | 0 | 1 | 0 | 1 | 0 | 0 | 1 | 0 | 0 | 1 | 0 | 1.7 | 0.7 |
| *Turdus viscivorus* | Ireland | 117.8 | 0 | 0 | 1 | 0 | 0 | 0 | 0 | 1 | 15.70 | 0 | 0 | 0 | 0 | 1 | 0 | 1 | 0 | 0 | 1 | 0 | 0 | 0 | 1 | 0.3 | 0.1 |
|  |  |  |  |  |  |  |  |  |  |  |  |  |  |  |  |  |  |  |  |  |  |  |  |  |  |  |  |
| *Aegithalos caudatus* | France | 8.5 | 0 | 1 | 0 | 0 | 0 | 0 | 1 | 0 | 7.5 | 1 | 0 | 0 | 0 | 0 | 1 | 0 | 1 | 0 | 1 | 0 | 0 | 1 | 0 | 1.7 | 1.0 |
| *Anthus trivialis* | France | 21.61 | 0 | 1 | 0 | 0 | 1 | 1 | 0 | 0 | 15.1 | 0 | 0 | 1 | 0 | 0 | 0 | 1 | 1 | 1 | 0 | 0 | 0 | 1 | 0 | 2.1 | 3.0 |
| *Linaria cannabina* | France | 18.95 | 1 | 0 | 0 | 0 | 1 | 0 | 0 | 0 | 13 | 0 | 0 | 0 | 1 | 0 | 0 | 1 | 1 | 0 | 0 | 1 | 0 | 0 | 1 | 0.5 | 0.0 |
| *Carduelis carduelis* | France | 15.45 | 1 | 0 | 0 | 0 | 0 | 0 | 0 | 1 | 15.8 | 0 | 0 | 0 | 0 | 1 | 0 | 1 | 1 | 0 | 0 | 1 | 0 | 1 | 0 | 1.0 | 0.4 |
| *Chloris chloris* | France | 27.65 | 1 | 0 | 0 | 0 | 0 | 0 | 0 | 1 | 16.65 | 0 | 0 | 0 | 1 | 0 | 0 | 1 | 1 | 0 | 0 | 1 | 0 | 1 | 0 | 3.3 | 3.2 |
| *Certhia brachydactyla* | France | 9.5 | 0 | 1 | 0 | 0 | 0 | 0 | 1 | 0 | 17.95 | 1 | 1 | 0 | 0 | 0 | 0 | 1 | 1 | 0 | 1 | 0 | 0 | 0 | 1 | 5.0 | 5.2 |
| *Cettia cetti* | France | 14.1 | 0 | 1 | 0 | 0 | 1 | 0 | 0 | 0 | 14.4 | 1 | 0 | 0 | 0 | 0 | 1 | 0 | 1 | 0 | 1 | 0 | 0 | 1 | 0 | 0.3 | 0.0 |
| *Columba palumbus* | France | 301 | 1 | 0 | 0 | 0 | 0 | 0 | 0 | 1 | 21.25 | 1 | 0 | 0 | 0 | 1 | 0 | 1 | 0 | 0 | 1 | 0 | 0 | 0 | 1 | 0.6 | 0.6 |
| *Corvus corone* | France | 484 | 0 | 0 | 1 | 0 | 1 | 0 | 0 | 0 | 55.1 | 0 | 0 | 0 | 0 | 1 | 0 | 1 | 0 | 0 | 1 | 0 | 0 | 0 | 1 | 1.3 | 0.9 |
| *Cuculus canorus* | France | 111.5 | 0 | 1 | 0 | 0 | 0 | 0 | 0 | 1 | 27.25 | 0 | 0 | 0 | 0 | 0 | 1 | 1 | 0 | 1 | 0 | 0 | 0 | 0 | 1 | 2.2 | 2.5 |
| *Cyanistes caeruleus* | France | 10.85 | 0 | 0 | 1 | 0 | 0 | 0 | 1 | 0 | 9.05 | 1 | 1 | 0 | 0 | 0 | 0 | 0 | 0 | 0 | 1 | 0 | 0 | 1 | 0 | 5.9 | 2.3 |
| *Dendrocopos major* | France | 89.7 | 0 | 1 | 0 | 0 | 0 | 0 | 1 | 0 | 28.9 | 1 | 1 | 0 | 0 | 0 | 0 | 1 | 1 | 0 | 1 | 0 | 0 | 0 | 1 | 2.7 | 3.7 |
| *Dendrocopos minor* | France | 20.68 | 0 | 1 | 0 | 0 | 0 | 0 | 1 | 0 | 17.3 | 1 | 1 | 0 | 0 | 0 | 0 | 1 | 0 | 0 | 1 | 0 | 0 | 0 | 1 | 1.0 | 0.5 |
| *Emberiza cirlus* | France | 25.6 | 1 | 0 | 0 | 0 | 1 | 0 | 0 | 0 | 14.15 | 1 | 0 | 0 | 1 | 0 | 0 | 1 | 1 | 0 | 1 | 0 | 1 | 0 | 0 | 0.2 | 0.2 |
| *Erithacus rubecula* | France | 18.25 | 0 | 0 | 1 | 0 | 1 | 0 | 0 | 0 | 14.3 | 0 | 0 | 1 | 0 | 0 | 0 | 1 | 1 | 0 | 1 | 0 | 1 | 0 | 0 | 5.5 | 5.7 |
| *Ficedula hypoleuca* | France | 14.9 | 0 | 1 | 0 | 1 | 0 | 0 | 0 | 0 | 11.8 | 1 | 1 | 0 | 0 | 0 | 0 | 1 | 1 | 1 | 0 | 0 | 1 | 0 | 0 | 0.1 | 0.0 |
| *Fringilla coelebs* | France | 21.4 | 0 | 0 | 1 | 0 | 1 | 0 | 0 | 0 | 15.05 | 1 | 0 | 0 | 0 | 1 | 0 | 1 | 0 | 0 | 0 | 1 | 0 | 0 | 1 | 6.8 | 9.6 |
| *Garrulus glandarius* | France | 161.7 | 0 | 0 | 1 | 0 | 0 | 0 | 0 | 1 | 33.2 | 1 | 0 | 0 | 0 | 1 | 0 | 1 | 0 | 0 | 1 | 0 | 0 | 0 | 1 | 1.3 | 1.5 |
| *Locustella naevia* | France | 13.1 | 0 | 1 | 0 | 0 | 0 | 1 | 0 | 0 | 14.3 | 0 | 0 | 1 | 0 | 0 | 0 | 1 | 1 | 1 | 0 | 0 | 0 | 1 | 0 | 0.1 | 0.4 |
| *Lophophanes cristatus* | France | 11.5 | 0 | 1 | 0 | 0 | 0 | 0 | 1 | 0 | 11.3 | 1 | 1 | 0 | 0 | 0 | 0 | 1 | 1 | 0 | 1 | 0 | 0 | 1 | 0 | 2.0 | 5.0 |
| *Luscinia megarhynchos* | France | 21.12 | 0 | 1 | 0 | 0 | 1 | 0 | 0 | 0 | 17.1 | 0 | 0 | 1 | 0 | 0 | 0 | 1 | 1 | 1 | 0 | 0 | 1 | 0 | 0 | 4.3 | 3.6 |
| *Motacilla alba* | France | 20.75 | 0 | 1 | 0 | 0 | 1 | 0 | 0 | 0 | 16.1 | 0 | 1 | 0 | 0 | 0 | 0 | 1 | 1 | 0 | 1 | 0 | 0 | 0 | 1 | 0.7 | 0.4 |
| *Muscicapa striata* | France | 14.7 | 0 | 1 | 0 | 1 | 0 | 0 | 0 | 0 | 15.45 | 0 | 0 | 0 | 0 | 0 | 1 | 1 | 1 | 1 | 0 | 0 | 1 | 0 | 0 | 1.0 | 0.2 |
| *Oriolus oriolus* | France | 67.8 | 0 | 1 | 0 | 0 | 0 | 0 | 1 | 0 | 27.8 | 1 | 0 | 0 | 0 | 1 | 0 | 1 | 0 | 1 | 0 | 0 | 0 | 0 | 1 | 0.4 | 0.0 |
| *Parus major* | France | 17.75 | 0 | 0 | 1 | 0 | 0 | 0 | 1 | 0 | 12.55 | 1 | 1 | 0 | 0 | 0 | 0 | 0 | 0 | 0 | 1 | 0 | 0 | 1 | 0 | 6.6 | 5.1 |
| *Periparus ater* | France | 9.7 | 0 | 0 | 1 | 0 | 0 | 0 | 1 | 0 | 11.15 | 1 | 1 | 0 | 0 | 0 | 0 | 0 | 1 | 0 | 1 | 0 | 1 | 0 | 0 | 0.2 | 0.0 |
| *Phoenicurus phoenicurus* | France | 15.9 | 0 | 1 | 0 | 1 | 1 | 0 | 1 | 0 | 14.6 | 1 | 1 | 0 | 0 | 0 | 0 | 1 | 1 | 1 | 0 | 0 | 0 | 1 | 0 | 0.6 | 0.0 |
| *Phylloscopus collybita* | France | 7.7 | 0 | 1 | 0 | 0 | 0 | 0 | 1 | 0 | 11.1 | 1 | 0 | 1 | 0 | 0 | 0 | 1 | 1 | 1 | 0 | 0 | 0 | 0 | 1 | 6.2 | 10.9 |
| *Pica pica* | France | 213 | 0 | 0 | 1 | 0 | 1 | 0 | 0 | 0 | 40.3 | 0 | 0 | 0 | 0 | 1 | 0 | 1 | 0 | 0 | 1 | 0 | 0 | 0 | 1 | 0.1 | 0.0 |
| *Picus viridis* | France | 189 | 0 | 1 | 0 | 0 | 1 | 0 | 0 | 0 | 45.5 | 0 | 1 | 0 | 0 | 0 | 0 | 1 | 0 | 0 | 1 | 0 | 0 | 0 | 1 | 2.0 | 1.7 |
| *Prunella modularis* | France | 18.6 | 0 | 0 | 1 | 0 | 1 | 0 | 0 | 0 | 14.65 | 1 | 0 | 0 | 1 | 0 | 0 | 1 | 1 | 0 | 1 | 0 | 1 | 0 | 0 | 0.5 | 0.2 |
| *Pyrrhula pyrrhula* | France | 21 | 1 | 0 | 0 | 0 | 0 | 0 | 1 | 0 | 15.35 | 1 | 0 | 0 | 0 | 1 | 0 | 1 | 1 | 0 | 1 | 0 | 0 | 1 | 0 | 0.0 | 0.2 |
| *Regulus ignicapillus* | France | 5.4 | 0 | 1 | 0 | 0 | 0 | 0 | 1 | 0 | 11.2 | 0 | 0 | 0 | 0 | 1 | 0 | 0 | 1 | 0 | 0 | 1 | 1 | 0 | 0 | 0.8 | 0.6 |
| *Saxicola torquata* | France | 14.9 | 0 | 1 | 0 | 0 | 1 | 0 | 0 | 0 | 15 | 1 | 0 | 1 | 0 | 0 | 0 | 1 | 0 | 0 | 0 | 1 | 0 | 1 | 0 | 0.8 | 1.1 |
| *Serinus serinus* | France | 11.8 | 1 | 0 | 0 | 0 | 1 | 1 | 0 | 0 | 10.3 | 0 | 0 | 0 | 0 | 1 | 0 | 1 | 1 | 0 | 0 | 1 | 1 | 0 | 0 | 0.5 | 0.2 |
| *Sitta europaea* | France | 23.4 | 1 | 1 | 0 | 0 | 0 | 0 | 1 | 0 | 19 | 1 | 1 | 0 | 0 | 0 | 0 | 0 | 1 | 0 | 1 | 0 | 0 | 1 | 0 | 2.8 | 1.7 |
| *Streptopelia turtur* | France | 132 | 1 | 0 | 0 | 0 | 1 | 0 | 0 | 0 | 16.8 | 0 | 0 | 0 | 1 | 0 | 0 | 1 | 1 | 1 | 0 | 0 | 0 | 0 | 1 | 0.9 | 0.8 |
| *Sturnus vulgaris* | France | 80.5 | 0 | 0 | 1 | 0 | 1 | 0 | 0 | 0 | 29.05 | 0 | 1 | 0 | 0 | 0 | 0 | 1 | 0 | 0 | 0 | 1 | 0 | 1 | 0 | 3.1 | 1.3 |
| *Sylvia atricapilla* | France | 18.6 | 0 | 0 | 1 | 0 | 0 | 0 | 1 | 0 | 14.45 | 1 | 0 | 0 | 1 | 0 | 0 | 1 | 1 | 1 | 0 | 0 | 0 | 1 | 0 | 6.6 | 7.5 |
| *Sylvia borin* | France | 19.05 | 0 | 0 | 1 | 0 | 0 | 1 | 0 | 0 | 14.5 | 1 | 0 | 0 | 1 | 0 | 0 | 1 | 1 | 1 | 0 | 0 | 1 | 0 | 0 | 0.1 | 0.0 |
| *Sylvia communis* | France | 16.15 | 0 | 0 | 1 | 0 | 0 | 1 | 0 | 0 | 13.2 | 1 | 0 | 0 | 1 | 0 | 0 | 1 | 1 | 1 | 0 | 0 | 1 | 0 | 0 | 0.3 | 0.6 |
| *Sylvia undata* | France | 9.4 | 0 | 1 | 0 | 0 | 0 | 1 | 0 | 0 | 12.8 | 1 | 0 | 0 | 1 | 0 | 0 | 1 | 1 | 0 | 1 | 0 | 1 | 0 | 0 | 0.1 | 0.1 |
| *Troglodytes troglodytes* | France | 8.9 | 0 | 1 | 0 | 0 | 0 | 1 | 0 | 0 | 13.65 | 0 | 0 | 1 | 0 | 0 | 0 | 1 | 1 | 0 | 1 | 0 | 1 | 0 | 0 | 8.3 | 10.8 |
| *Turdus merula* | France | 95.85 | 0 | 0 | 1 | 0 | 0 | 0 | 0 | 1 | 17.2 | 0 | 0 | 0 | 1 | 0 | 0 | 1 | 0 | 0 | 0 | 1 | 0 | 1 | 0 | 4.9 | 3.4 |
| *Turdus philomelos* | France | 70.5 | 0 | 0 | 1 | 0 | 1 | 0 | 0 | 0 | 14.25 | 0 | 0 | 0 | 0 | 1 | 0 | 1 | 0 | 0 | 1 | 0 | 0 | 1 | 0 | 3.8 | 2.5 |
| *Turdus viscivorus* | France | 117.8 | 0 | 0 | 1 | 0 | 0 | 0 | 0 | 1 | 15.7 | 0 | 0 | 0 | 0 | 1 | 0 | 1 | 0 | 0 | 1 | 0 | 0 | 0 | 1 | 0.5 | 1.7 |
|  |  |  |  |  |  |  |  |  |  |  |  |  |  |  |  |  |  |  |  |  |  |  |  |  |  |  |  |
| *Aegithalos caudatus* | Portugal | 8.5 | 0 | 1 | 0 | 0 | 0 | 0 | 1 | 0 | 7.5 | 1 | 0 | 0 | 0 | 0 | 1 | 0 | 1 | 0 | 1 | 0 | 0 | 1 | 0 | 4.0 | 0.3 |
| *Alauda arvensis* | Portugal | 26.55 | 0 | 0 | 1 | 0 | 1 | 0 | 0 | 0 | 15.2 | 1 | 0 | 1 | 0 | 0 | 0 | 1 | 1 | 0 | 0 | 1 | 1 | 0 | 0 | 0.2 | 0.0 |
| *Linaria cannabina* | Portugal | 18.95 | 1 | 0 | 0 | 0 | 1 | 0 | 0 | 0 | 13 | 0 | 0 | 0 | 1 | 0 | 0 | 1 | 1 | 0 | 0 | 1 | 0 | 0 | 1 | 1.7 | 7.2 |
| *Chloris chloris* | Portugal | 27.65 | 1 | 0 | 0 | 0 | 0 | 0 | 0 | 1 | 16.65 | 0 | 0 | 0 | 1 | 0 | 0 | 1 | 1 | 0 | 0 | 1 | 0 | 1 | 0 | 0.4 | 10.2 |
| *Certhia brachydactyla* | Portugal | 9.5 | 0 | 1 | 0 | 0 | 0 | 0 | 1 | 0 | 17.95 | 1 | 1 | 0 | 0 | 0 | 0 | 1 | 1 | 0 | 1 | 0 | 0 | 0 | 1 | 4.1 | 3.9 |
| *Cisticola juncidis* | Portugal | 8.4 | 0 | 1 | 0 | 0 | 0 | 1 | 0 | 0 | 12.3 | 0 | 0 | 0 | 1 | 0 | 0 | 1 | 1 | 0 | 1 | 0 | 0 | 1 | 0 | 0.0 | 0.3 |
| *Columba livia* | Portugal | 340 | 0 | 0 | 1 | 0 | 1 | 0 | 0 | 0 | 18.8 | 0 | 0 | 0 | 0 | 0 | 1 | 1 | 0 | 0 | 1 | 0 | 0 | 0 | 1 | 0.0 | 0.5 |
| *Columba palumbus* | Portugal | 301 | 1 | 0 | 0 | 0 | 0 | 0 | 0 | 1 | 21.25 | 1 | 0 | 0 | 0 | 1 | 0 | 1 | 0 | 0 | 1 | 0 | 0 | 0 | 1 | 0.0 | 0.1 |
| *Corvus corone* | Portugal | 484 | 0 | 0 | 1 | 0 | 1 | 0 | 0 | 0 | 55.1 | 0 | 0 | 0 | 0 | 1 | 0 | 1 | 0 | 0 | 1 | 0 | 0 | 0 | 1 | 0.0 | 2.2 |
| *Cyanistes caeruleus* | Portugal | 10.85 | 0 | 0 | 1 | 0 | 0 | 0 | 1 | 0 | 9.05 | 1 | 1 | 0 | 0 | 0 | 0 | 0 | 0 | 0 | 1 | 0 | 0 | 1 | 0 | 8.6 | 1.4 |
| *Dendrocopos major* | Portugal | 89.7 | 0 | 1 | 0 | 0 | 0 | 0 | 1 | 0 | 28.9 | 1 | 1 | 0 | 0 | 0 | 0 | 1 | 1 | 0 | 1 | 0 | 0 | 0 | 1 | 0.2 | 1.4 |
| *Emberiza cia* | Portugal | 23.9 | 1 | 0 | 0 | 0 | 1 | 0 | 0 | 0 | 14 | 1 | 0 | 0 | 1 | 0 | 0 | 1 | 1 | 0 | 1 | 0 | 1 | 0 | 0 | 2.4 | 1.3 |
| *Erithacus rubecula* | Portugal | 18.25 | 0 | 0 | 1 | 0 | 1 | 0 | 0 | 0 | 14.3 | 0 | 0 | 1 | 0 | 0 | 0 | 1 | 1 | 0 | 1 | 0 | 1 | 0 | 0 | 8.6 | 6.7 |
| *Ficedula hypoleuca* | Portugal | 14.9 | 0 | 1 | 0 | 1 | 0 | 0 | 0 | 0 | 11.8 | 1 | 1 | 0 | 0 | 0 | 0 | 1 | 1 | 1 | 0 | 0 | 1 | 0 | 0 | 0.9 | 0.2 |
| *Fringilla coelebs* | Portugal | 21.4 | 0 | 0 | 1 | 0 | 1 | 0 | 0 | 0 | 15.05 | 1 | 0 | 0 | 0 | 1 | 0 | 1 | 0 | 0 | 0 | 1 | 0 | 0 | 1 | 7.5 | 4.2 |
| *Garrulus glandarius* | Portugal | 161.7 | 0 | 0 | 1 | 0 | 0 | 0 | 0 | 1 | 33.2 | 1 | 0 | 0 | 0 | 1 | 0 | 1 | 0 | 0 | 1 | 0 | 0 | 0 | 1 | 2.1 | 2.4 |
| *Lophophanes cristatus* | Portugal | 11.5 | 0 | 1 | 0 | 0 | 0 | 0 | 1 | 0 | 11.3 | 1 | 1 | 0 | 0 | 0 | 0 | 1 | 1 | 0 | 1 | 0 | 0 | 1 | 0 | 9.6 | 9.0 |
| *Oriolus oriolus* | Portugal | 67.8 | 0 | 1 | 0 | 0 | 0 | 0 | 1 | 0 | 27.8 | 1 | 0 | 0 | 0 | 1 | 0 | 1 | 0 | 1 | 0 | 0 | 0 | 0 | 1 | 0.0 | 0.7 |
| *Parus major* | Portugal | 17.75 | 0 | 0 | 1 | 0 | 0 | 0 | 1 | 0 | 12.55 | 1 | 1 | 0 | 0 | 0 | 0 | 0 | 0 | 0 | 1 | 0 | 0 | 1 | 0 | 0.8 | 3.2 |
| *Passer domesticus* | Portugal | 30.2 | 0 | 0 | 1 | 0 | 0 | 0 | 0 | 1 | 15.6 | 0 | 1 | 0 | 0 | 0 | 0 | 1 | 0 | 0 | 1 | 0 | 1 | 0 | 0 | 0.0 | 2.8 |
| *Periparus ater* | Portugal | 9.7 | 0 | 0 | 1 | 0 | 0 | 0 | 1 | 0 | 11.15 | 1 | 1 | 0 | 0 | 0 | 0 | 0 | 1 | 0 | 1 | 0 | 1 | 0 | 0 | 13.4 | 11.5 |
| *Phylloscopus collybita* | Portugal | 7.7 | 0 | 1 | 0 | 0 | 0 | 0 | 1 | 0 | 11.1 | 1 | 0 | 1 | 0 | 0 | 0 | 1 | 1 | 1 | 0 | 0 | 0 | 0 | 1 | 0.0 | 0.2 |
| *Phylloscopus ibericus* | Portugal | 8.2 | 0 | 1 | 0 | 0 | 0 | 1 | 0 | 0 | 11.2 | 0 | 0 | 1 | 0 | 0 | 0 | 1 | 1 | 1 | 0 | 0 | 1 | 0 | 0 | 0.8 | 0.0 |
| *Picus sharpei* | Portugal | 189 | 0 | 1 | 0 | 0 | 1 | 0 | 0 | 0 | 45.5 | 0 | 1 | 0 | 0 | 0 | 0 | 1 | 0 | 0 | 1 | 0 | 0 | 0 | 1 | 2.3 | 4.2 |
| *Prunella modularis* | Portugal | 18.6 | 0 | 0 | 1 | 0 | 1 | 0 | 0 | 0 | 14.65 | 1 | 0 | 0 | 1 | 0 | 0 | 1 | 1 | 0 | 1 | 0 | 1 | 0 | 0 | 3.8 | 1.0 |
| *Pyrrhula pyrrhula* | Portugal | 21 | 1 | 0 | 0 | 0 | 0 | 0 | 1 | 0 | 15.35 | 1 | 0 | 0 | 0 | 1 | 0 | 1 | 1 | 0 | 1 | 0 | 0 | 1 | 0 | 2.1 | 0.6 |
| *Regulus ignicapillus* | Portugal | 5.4 | 0 | 1 | 0 | 0 | 0 | 0 | 1 | 0 | 11.2 | 0 | 0 | 0 | 0 | 1 | 0 | 0 | 1 | 0 | 0 | 1 | 1 | 0 | 0 | 2.7 | 2.6 |
| *Saxicola torquata* | Portugal | 14.9 | 0 | 1 | 0 | 0 | 1 | 0 | 0 | 0 | 15 | 1 | 0 | 1 | 0 | 0 | 0 | 1 | 0 | 0 | 0 | 1 | 0 | 1 | 0 | 0.0 | 2.0 |
| *Serinus serinus* | Portugal | 11.8 | 1 | 0 | 0 | 0 | 1 | 1 | 0 | 0 | 10.3 | 0 | 0 | 0 | 0 | 1 | 0 | 1 | 1 | 0 | 0 | 1 | 1 | 0 | 0 | 0.2 | 1.8 |
| *Sitta europaea* | Portugal | 23.4 | 1 | 1 | 0 | 0 | 0 | 0 | 1 | 0 | 19 | 1 | 1 | 0 | 0 | 0 | 0 | 0 | 1 | 0 | 1 | 0 | 0 | 1 | 0 | 2.2 | 0.0 |
| *Streptopelia decaocto* | Portugal | 196.5 | 0 | 0 | 1 | 0 | 1 | 0 | 0 | 0 | 16.75 | 0 | 0 | 0 | 0 | 1 | 0 | 1 | 0 | 0 | 1 | 0 | 0 | 0 | 1 | 0.0 | 0.5 |
| *Sylvia atricapilla* | Portugal | 18.6 | 0 | 0 | 1 | 0 | 0 | 0 | 1 | 0 | 14.45 | 1 | 0 | 0 | 1 | 0 | 0 | 1 | 1 | 1 | 0 | 0 | 0 | 1 | 0 | 4.5 | 4.9 |
| *Sylvia undata* | Portugal | 9.4 | 0 | 1 | 0 | 0 | 0 | 1 | 0 | 0 | 12.8 | 1 | 0 | 0 | 1 | 0 | 0 | 1 | 1 | 0 | 1 | 0 | 1 | 0 | 0 | 1.6 | 0.6 |
| *Troglodytes troglodytes* | Portugal | 8.9 | 0 | 1 | 0 | 0 | 0 | 1 | 0 | 0 | 13.65 | 0 | 0 | 1 | 0 | 0 | 0 | 1 | 1 | 0 | 1 | 0 | 1 | 0 | 0 | 12.1 | 8.2 |
| *Turdus merula* | Portugal | 95.85 | 0 | 0 | 1 | 0 | 0 | 0 | 0 | 1 | 17.2 | 0 | 0 | 0 | 1 | 0 | 0 | 1 | 0 | 0 | 0 | 1 | 0 | 1 | 0 | 2.9 | 3.3 |
| *Turdus philomelos* | Portugal | 70.5 | 0 | 0 | 1 | 0 | 1 | 0 | 0 | 0 | 14.25 | 0 | 0 | 0 | 0 | 1 | 0 | 1 | 0 | 0 | 1 | 0 | 0 | 1 | 0 | 0.0 | 0.1 |
| *Turdus viscivorus* | Portugal | 117.8 | 0 | 0 | 1 | 0 | 0 | 0 | 0 | 1 | 15.7 | 0 | 0 | 0 | 0 | 1 | 0 | 1 | 0 | 0 | 1 | 0 | 0 | 0 | 1 | 0.1 | 0.3 |
