# Supplementary material for: Functional shifts in bird communities from semi-natural oak forests to conifer plantations are not consistent across Europe
Source: PLoS One. 2019 Jul 22;14(7):e0220155. doi: 10.1371/journal.pone.0220155 (PMC6645557; doi:10.1371/journal.pone.0220155)
Supplement: S3 Table — (DOCX) [file pone.0220155.s003.docx]

**S3 Table.** Fourth corner test statistics

|  |  | Oak Ireland | | Conifer Ireland | | Oak France | | Conifer France | | Oak Portugal | | Conifer Portugal | |
| --- | --- | --- | --- | --- | --- | --- | --- | --- | --- | --- | --- | --- | --- |
| Trait group | Traits | Obs | P-value | Obs | P-value | Obs | P-value | Obs | P-value | Obs | P-value | Obs | P-value |
| Body size | mass | 0.096 | 0.003 | -0.096 | 0.003 | 0.038 | 0.013 | -0.038 | 0.013 | -0.165 | 0.000 | 0.165 | 0.000 |
| Diet | herbivore | 0.046 | 0.058 | -0.046 | 0.058 | 0.044 | 0.025 | -0.044 | 0.025 | -0.171 | 0.011 | 0.171 | 0.011 |
|  | insectivore | -0.128 | 0.103 | 0.128 | 0.103 | -0.077 | 0.000 | 0.077 | 0.000 | 0.071 | 0.238 | -0.071 | 0.238 |
|  | mixed | 0.119 | 0.126 | -0.119 | 0.126 | 0.067 | 0.001 | -0.067 | 0.001 | 0.075 | 0.212 | -0.075 | 0.212 |
| Foraging | air | 0.033 | 0.001 | -0.033 | 0.001 | 0.080 | 0.000 | -0.080 | 0.000 | 0.047 | 0.728 | -0.047 | 0.728 |
|  | ground | 0.067 | 0.258 | -0.067 | 0.258 | 0.027 | 0.144 | -0.027 | 0.144 | -0.054 | 0.405 | 0.054 | 0.405 |
|  | lower vegetation | 0.013 | 0.800 | -0.013 | 0.800 | -0.071 | 0.000 | 0.071 | 0.000 | 0.055 | 0.285 | -0.055 | 0.285 |
|  | upper vegetation | -0.114 | 0.085 | 0.114 | 0.085 | 0.002 | 0.903 | -0.002 | 0.903 | 0.126 | 0.037 | -0.126 | 0.037 |
|  | mixed | 0.105 | 0.002 | -0.105 | 0.002 | 0.012 | 0.532 | -0.012 | 0.532 | -0.211 | 0.000 | 0.211 | 0.000 |
| Bill length | average | 0.080 | 0.085 | -0.080 | 0.085 | 0.037 | 0.019 | -0.037 | 0.019 | -0.163 | 0.001 | 0.163 | 0.001 |
| Habitat | specialist | -0.115 | 0.077 | 0.115 | 0.077 | -0.037 | 0.035 | 0.037 | 0.035 | 0.193 | 0.002 | -0.193 | 0.002 |
| Nest location | cavity | 0.033 | 0.705 | -0.033 | 0.705 | 0.075 | 0.000 | -0.075 | 0.000 | 0.048 | 0.414 | -0.048 | 0.414 |
|  | ground | 0.074 | 0.235 | -0.074 | 0.235 | -0.100 | 0.000 | 0.100 | 0.000 | 0.058 | 0.302 | -0.058 | 0.302 |
|  | shrub | 0.076 | 0.058 | -0.076 | 0.058 | 0.016 | 0.433 | -0.016 | 0.433 | -0.138 | 0.030 | 0.138 | 0.030 |
|  | tree | -0.150 | 0.074 | 0.150 | 0.074 | -0.005 | 0.792 | 0.005 | 0.792 | -0.013 | 0.814 | 0.013 | 0.814 |
|  | mixed | 0.115 | 0.002 | -0.115 | 0.002 | 0.041 | 0.026 | -0.041 | 0.026 | 0.102 | 0.463 | -0.102 | 0.463 |
| Clutch size | small/medium | 0.158 | 0.034 | -0.158 | 0.034 | -0.127 | 0.000 | 0.127 | 0.000 | -0.146 | 0.018 | 0.146 | 0.018 |
| Life span | short | -0.234 | 0.001 | 0.234 | 0.001 | -0.079 | 0.000 | 0.079 | 0.000 | 0.041 | 0.495 | -0.041 | 0.495 |
| Migration | migratory | 0.063 | 0.082 | -0.063 | 0.082 | -0.055 | 0.001 | 0.055 | 0.001 | 0.003 | 0.950 | -0.003 | 0.950 |
|  | resident | -0.099 | 0.049 | 0.099 | 0.049 | 0.030 | 0.095 | -0.030 | 0.095 | 0.171 | 0.006 | -0.171 | 0.006 |
|  | mixed | 0.076 | 0.085 | -0.076 | 0.085 | 0.023 | 0.204 | -0.023 | 0.204 | -0.186 | 0.003 | 0.186 | 0.003 |
| Range | small | -0.288 | 0.001 | 0.288 | 0.001 | -0.007 | 0.701 | 0.007 | 0.701 | 0.101 | 0.086 | -0.101 | 0.086 |
|  | medium | 0.305 | 0.001 | -0.305 | 0.001 | 0.077 | 0.000 | -0.077 | 0.000 | -0.001 | 0.980 | 0.001 | 0.980 |
|  | large | 0.080 | 0.077 | -0.080 | 0.077 | -0.073 | 0.000 | 0.073 | 0.000 | -0.118 | 0.052 | 0.118 | 0.052 |
