# Supplementary material for: Functional shifts in bird communities from semi-natural oak forests to conifer plantations are not consistent across Europe
Source: PLoS One. 2019 Jul 22;14(7):e0220155. doi: 10.1371/journal.pone.0220155 (PMC6645557; doi:10.1371/journal.pone.0220155)
Supplement: S4 Table — (DOCX) [file pone.0220155.s004.docx]

**S4 Table.** Test statistics for differences in diversity metrics between semi-natural oak forest and conifer plantations (Mann-Whitney U test). Results in bold indicate where a test found significant differences between the two forest types.

| Diversity measure | Ireland | | France | | Portugal | |
| --- | --- | --- | --- | --- | --- | --- |
|  | Statistic | P-value | Statistic | P-value | Statistic | P-value |
| Species richness | **65** | **0.004** | **2210** | **<0.001** | 42 | 0.928 |
| Shannon index | **70** | **<0.001** | **2169** | **<0.001** | 42 | 0.931 |
| Rao’s Quadratic Entropy | **70** | **<0.001** | **1931** | **<0.001** | **16** | **0.031** |
| Functional dispersion | **70** | **<0.001** | **1900** | **<0.001** | **15** | **0.024** |
| Phylogenetic diversity | **64** | **0.003** | **2176** | **<0.001** | 49 | 0.489 |
| Phylogenetic dispersion | 18 | 0.109 | **763** | **0.001** | 25 | 0.190 |
