# Supplementary material for: Functional shifts in bird communities from semi-natural oak forests to conifer plantations are not consistent across Europe
Source: PLoS One. 2019 Jul 22;14(7):e0220155. doi: 10.1371/journal.pone.0220155 (PMC6645557; doi:10.1371/journal.pone.0220155)
Supplement: S1 File — (DOCX) [file pone.0220155.s005.docx]

**S1** **File**: Point count sampling methodology

In all study regions point count survey methodology followed Bibby [1]. All birds heard and seen were recorded, excluding data relative to birds of prey, owls, swifts, swallows and nightjars from the analyses. Surveys were cancelled on days with strong wind and rain. In Ireland, birds were surveyed twice, in April to May (first visit) and May to June (second visit), during the breeding seasons of 2007 and 2008 [2]. In each forest patch, six points were randomly placed a minimum of 100 m apart in edge and interior forest habitat. Point counts lasted for 10 min within 50 m distance from the observer. Counts were conducted either during the morning (800-1100h) or during the afternoon (1400-1700h).

In France, point counts were also conducted over two visits, in early April to mid-May (first visit) and mid-May to June (second visit), during the breeding seasons of 2001 to 2003 [3]. Two observers performed the surveys and permutated first and second visits for a given point to avoid a possible observer effect. Points were placed at least 400 m apart. Point counts lasted 20 min, using a semi-quantitative abundance index where a territorial male or pair was noted as 1 and a non-singing bird was noted as 0.5. The final abundance index was the maximum score obtained for each species among the two visits. Point counts were performed within 5 h after sunrise.

In Portugal, bird communities were surveyed during the first 3 h of the day, during July and August of 2005 [4]. The sampling effort was proportional to a species-area relationship across forest patches. Each point count lasted 15 min, within 25 m radius. Abundances of each species were averaged for each forest patch, to give one sample per forest patch for use in analysis.

For the Ireland dataset, distance sampling was used to estimate the probability of detecting a bird with distance from observer, and these used to convert counts to estimates of density [2]. In France and Portugal raw abundances were used as distance of detections was not recorded. This means that the abundance of difficult to observe species is likely to be underestimated in France and Portugal relative to Ireland. For the France dataset, the use of a semi-quantitative index to give more weight to singing individuals more likely to be using the habitat means this dataset will be weighted more heavily to acoustic detections. Due to these differences in survey designs and timings the abundance data are not directly comparable between the three regions. We therefore quantitatively analyse abundance data for each region separately, and focus on more qualitative assessments for between region comparisons.

**References**

1. Bibby CJ, Burgess ND, Hill DA. Bird census techniques. London: Academic Press; 2000.

2. Sweeney OFM, Wilson MW, Irwin S, Kelly TC, O'Halloran J. Are bird density, species richness and community structure similar between native woodlands and non-native plantations in an area with a generalist bird fauna? Biodiversity and Conservation. 2010;19(8):2329-42.

3. Barbaro L, van Halder I. Linking bird, carabid beetle and butterfly life-history traits to habitat fragmentation in mosaic landscapes. Ecography. 2009;32(2):321-33.

4. Proença VM, Pereira HM, Guilherme J, Vicente L. Plant and bird diversity in natural forests and in native and exotic plantations in NW Portugal. Acta Oecol-Int J Ecol. 2010;36(2):219-26
